# Magnesium in Type 2 Diabetes Mellitus, Obesity, and Metabolic Syndrome

**DOI:** 10.3390/nu14030714

**Published:** 2022-02-08

**Authors:** Mario Barbagallo, Nicola Veronese, Ligia J. Dominguez

**Affiliations:** 1 Department PROMISE, University of Palermo, 90127 Palermo, Italy; nicola.veronese@unipa.it (N.V.); ligia.dominguez@unipa.it (L.J.D.); 2 Faculty of Medicine, Kore University, 94100 Enna, Italy

Metabolic syndrome is a constellation of risk factors, including obesity, hypertension, insulin resistance, and altered lipid profile, which, if left untreated, will often progress to type 2 diabetes, which frequently complicates the syndrome. In each of these conditions, a magnesium (Mg) deficit has been consistently found [1]. Conversely, subjects with Mg deficiency are at a higher risk of developing any of these metabolic alterations. It has been suggested that a chronic low-grade inflammation triggered or exacerbated by a Mg deficit may favour this cluster formation. Thus, a low Mg status is highly prevalent in several pathological conditions characterized by having a chronic inflammatory component. It is important to mention that even a subclinical Mg deficit may cause the production of inflammatory cytokines and free radicals, inducing a chronic low-grade inflammation status [1]. This chronic state of inflammation is a common substrate for several non-communicable diseases, including hypertension, cardiovascular disorders, metabolic syndrome, and type 2 diabetes mellitus [2,3]. Obesity is also characterized by an increased inflammatory status, being itself a cardiovascular and metabolic risk factor.

Mg deprivation may trigger chronic inflammation directly, but also indirectly by altering the intestinal microbiota. A number of studies have defined a possible role for the gut microbiota in the pathogenesis of the metabolic syndrome, while diets mostly based on refined foods not only are poor in Mg content but also negatively alter the gut microbial ecosystem. Existing recent literature supports the gut microbiome’s potential influence on the various risk factors of metabolic syndrome. Interactions between gut microbiota and host metabolism have been shown to be mediated by a number of factors, including inflammation. Thus, a Mg deficiency status, exacerbating chronic inflammatory stress already present in many pathological conditions, may trigger a vicious circle significantly contributing these conditions to cluster [1].

Mg deficiency in type 2 diabetes mellitus and metabolic syndrome, which may take the form of a latent subclinical Mg deficiency rather than a less common overt hypomagnesemia, may have physiopathological and clinical importance because the Mg ion is a critical cofactor for many enzymatic reactions involved in a myriad of metabolic processes. In the human body, Mg is the fourth most abundant mineral after calcium, sodium, and potassium, second only to potassium as an intracellular cation, being, however, the most common cellular divalent cation [2].

Mg is a crucial cofactor for hundreds of enzymatic reactions and biological processes (estimated now at over 600!) acting on the enzymes both, as a structural or catalytic component, and on the substrates; it is required for all the oxidative phosphorylation processes, energy production reactions, protein synthesis, glycolysis, and nucleic acid synthesis and stability [4]. In particular, cellular Mg is a necessary cofactor for numerous processes involved in carbohydrate metabolism. In particular, for its key presence in the Mg-ATP complex, being an essential factor for all of the rate-limiting enzymes of glycolysis, Mg regulates the activity of all enzymes involved in phosphorylation reactions.

Mg concentration is critical in the phosphorylation of insulin receptor tyrosine-kinase as well as all other protein kinases in the cellular insulin signalling, and all ATP and phosphate transfer-associated enzymes. Thus, a Mg deficit would cause an impairment of insulin receptor tyrosine-kinase’ activity, triggering post-receptorial insulin resistance and decreased cellular glucose utilization, that is, the lower the basal Mg, the greater the amount of insulin required to metabolize the same glucose load, indicating a decreased insulin sensitivity [2,3,4,5].

An impairment of the cellular Mg uptake mechanism and a decrease in the cellular ATP level may contribute, at least in part, to explain the decrease in cellular Mg content observed under diabetic conditions. A low Mg status itself is associated with a significant impairment of insulin-mediated glucose uptake and with a considerably increased risk of developing glucose intolerance and diabetes [2,3,4,5].

In western countries, it has been reported that around two-thirds of the adult population do not consume the estimated average requirement for Mg, causing a chronic marginal to moderate Mg deficit. Thus, modern Western diets contain a tremendous amount of refined grains and highly processed food; moreover, it has been calculated that around 80–90% of Mg would be lost during food cooking and processing. This dietary Mg scarcity is accentuated by the fact that, in the last few decades, the soil used for agriculture, is becoming increasingly deficient in essential minerals, particularly in Mg. It has been estimated that in the past sixty years, the Mg content in vegetables and fruit has decreased by 20–30% [6].

An increased Mg urinary wasting may also favor the Mg depletion in diabetes mellitus, while absorption and retention of dietary Mg seems not to be impaired in type 2 diabetic patients. Hyperglycemia and hyperinsulinemia may both have a role in the increased urinary Mg excretion contributing to Mg depletion. In this context, a vicious circle is exacerbated by the role of Mg deficiency to sustain a preexisting inflammatory status [7]. Several studies have demonstrated that Mg deprivation would cause elevation of several proinflammatory molecules, including tumor necrosis factor-alpha, interleukin (IL)-1-beta, IL-6, vascular cell adhesion protein-1, and plasminogen activator inhibitor-1; increased circulating inflammatory cells; and elevation of hepatic production and release of acute phase proteins (i.e., alfa2-macroglobulin, complement, and fibrinogen) [2,3].

The critical importance of altered Mg status to directly promote inflammatory cytokine release and tissutal insulin resistance also emphasizes the possible role of a chronic Mg deficiency status to increase the risk of developing type 2 diabetes and cardio-metabolic conditions, and, at the same time, to contribute to the clinical coincidence of apparently disparate clinical conditions characterized by altered insulin sensitivity, such as hypertension, obesity, metabolic syndrome, and type 2 diabetes [2,3,4,5].

Experimental models have shown that a chronic Mg insufficiency is associated with impaired post-receptorial function, consequently reducing glucose utilization in cells. Mg may have an action in improving insulin secretion from pancreatic beta cells, while the main action of Mg seems to be linked to a decrease in insulin resistance as shown by the improvements in the Homeostatic Model Assessment for Insulin Resistance (HOMA-IR) index, particularly in obese subjects and in subjects with metabolic syndrome, at high risk of diabetes, possibly implying that Mg may have a better action when a deposit of insulin is present [2,3,4,5]. This would suggest that Mg supplements may be more effective in patients with insulin resistance and hyperinsulinemia, such as subjects with obesity, metabolic syndrome and type 2 diabetes mellitus, while Mg action in type 1 diabetes mellitus subjects needs to be better clarified.

Mg action to improve insulin sensitivity is confirmed by a number of studies showing that Mg is able to decrease oxidative stress and inflammatory parameters, two main contributors of insulin resistance. It has been suggested that Mg may also have some additional positive effects on other parameters that may be linked to glucose metabolism, such as body composition, sleep quality and general health [2].

Plasma total Mg level (magnesemia) is a feasible and inexpensive (although not very sensitive) method to measure Mg status in routine clinical practice. However, Mg is mostly an intracellular ion; blood serums only contain less than 1% of the total body Mg. Approximately 24 g (1 mole) of Mg are present in the human body, of which almost two-thirds are stored in the bone and one third in the cellular compartment. Serum magnesemia is not a reliable index of global body Mg status because it declines only when the tissue deposits are depleted, and thus is unable to detect most of the mild to moderate Mg deficits. Because of this, Mg is still considered a “*forgotten ion*”, and Mg deficits are often underestimated and unrecognized. In addition, subclinical Mg deficits symptoms are generally aspecific and not easily correlated to the undetected electrolyte alteration by the clinician [2,3].

The reference range for serum Mg levels is 0.75–0.95 mmol/L (1.7–2.5 mg/dL or 1.5–1.9 meq/L), while frank hypomagnesemia is usually considered as serum Mg level lower than 0.7 mmol/L. Mg serum levels are extremely constant and very tightly controlled and are kept in a narrow range by the kidney and the small intestine, increasing their fractional Mg absorption in Mg deficiency conditions. Although no hormone is specific for Mg metabolism, Mg exchange between extracellular, cellular and skeletal compartments is regulated by several hormonal factors. Parathyroid hormone, insulin, vitamin D, calcitonin, and estrogens are the principal hormonal systems implicated in Mg metabolism. The kidney is the principal site of Mg excretion. Mg is essential for vitamin D synthesis and activation [4]. To further complicate the picture, obese and type 2 diabetic subjects are often deficient in vitamin D.

At the cellular level, Mg metabolism is regulated by membrane channels and transporters. Among them, some are ubiquitously expressed, such as transient receptor potential melastatin (TRPM) 7, Mg transporter 1 (MagT1), and solute carrier family 41 member 1 (SLC41A1). Others are tissue-specific, such as TRPM6, present in the kidney and colon, cyclin and CBS domain divalent metal cation transport mediator cyclin M2 (CNNM2), present in the kidney, and CNNM4, in the colon.

Low serum Mg (i.e., extracellular) triggers Mg transporters such as TRPM7 and SLC41A1, inducing Mg efflux from cells in the attempt to normalize serum Mg levels. Low intracellular Mg concentrations may trigger Mg stores in the mitochondria to release Mg through SLC41A3. Reduced mitochondrial Mg concentrations may alter Mg and ATP-associated mitochondrial signaling and functions. This may explain the mitochondrial excess production of oxidative stress and decreased ATP observed in Mg deficient experimental mice.

Intracellular free Mg levels and ionized Mg have been consistently found to be reduced in subjects with type 2 diabetes mellitus [8]. Ionized Mg has been suggested to be more sensitive than total Mg concentrations to detect subclinical Mg deficiencies, and it also has been found to be reduced in type 2 diabetes mellitus [9].

The role of poor dietary Mg content and the reduced body Mg status in facilitating the clustering of cardio-metabolic disorders in a deadly constellation would suggest a potential benefit of Mg supplementation in preventing or treating these conditions.

A recent umbrella review of systematic reviews with meta-analyses of observational studies and randomized controlled trials (RCTs) using placebo/no intervention as the control group have confirmed that higher Mg intakes are associated with a decreased risk of developing type 2 diabetes in different populations [10]. Mg beneficial actions may be mediated both through a direct effect on metabolic pathways or through an indirect effect linked to an anti-inflammatory action [11]. However, several issues are still to be clarified to better understand if Mg supplementation would be able to correct this vicious loop. A former study showed that oral magnesium supplementation improved insulin sensitivity and metabolic control in type 2 diabetic subjects [12]. A recent systematic review and meta-analysis of double-blind RCTs found that Mg supplementation appears to have a beneficial role and improves glucose parameters in people with type 2 diabetes. In obese subjects and or in subjects with glucose intolerance, at high risk of diabetes, Mg supplementation significantly improved plasma glucose, and insulin-sensitivity parameters [13].

The benefits of Mg supplements on glycemic profile have been found in many, but not all, studies. Discrepancies in baseline Mg levels and metabolic control may help to explain the differences among diverse studies. It is also undefined if Mg supplementation may have the same advantage of increasing dietary Mg allowance in food, if the different Mg salts may provide all of the same benefits, or if a particular Mg salt may have any specific action, hence being more beneficial. In addition, the different dosages to be used for any specific Mg salt to guarantee better outcomes need to be better defined.

The high Mg content in whole grains may also, at least in part, explain the favorable effect of a healthy diet that is abundant in fiber and whole grains on insulin sensitivity. The positive effects of a sufficiently elevated Mg intake on systemic inflammation and gut microbiota may also help to explain the favorable effects to improve insulin sensitivity and reduce the risk of developing type 2 diabetes mellitus.

Considering the worldwide prevalence of obesity, type 2 diabetes mellitus, and the metabolic syndrome constellation, the correction of wrong dietary habits and, eventually, the use of Mg supplements may be an inexpensive but valuable tool to be considered in order to contain the occurrence and the progression of these conditions, a hypothesis that needs to be confirmed by specific and well-designed trials with Mg.

Future prospective studies are thus still needed to support the potential role of dietary Mg supplementation as a possible public health strategy to reduce cardio-metabolic risk in the general population.
